# Historical human activities reshape evolutionary trajectories across both native and introduced ranges

**DOI:** 10.1002/ece3.6391

**Published:** 2020-05-24

**Authors:** Anthony L. Einfeldt, Linley K. Jesson, Jason A. Addison

**Affiliations:** ^1^ Department of Biology University of New Brunswick Fredericton NB Canada; ^2^ Department of Biology Dalhousie University Halifax NS Canada; ^3^ New Zealand Institute for Plant & Food Research Limited Auckland New Zealand

**Keywords:** anthropocene, evolution, human impacts, introduced species

## Abstract

The same vectors that introduce species to new ranges could move them among native populations, but how human‐mediated dispersal impacts native ranges has been difficult to address because human‐mediated dispersal and natural dispersal can simultaneously shape patterns of gene flow. Here, we disentangle human‐mediated dispersal from natural dispersal by exploiting a system where the primary vector was once extensive but has since ceased. From 10th to 19th Centuries, ships in the North Atlantic exchanged sediments dredged from the intertidal for ballast, which ended when seawater ballast tanks were adopted. We investigate genetic patterns from RADseq‐derived SNPs in the amphipod *Corophium volutator* (*n* = 121; 4,870 SNPs) and the annelid *Hediste diversicolor* (*n* = 78; 3,820 SNPs), which were introduced from Europe to North America, have limited natural dispersal capabilities, are abundant in intertidal sediments, but not commonly found in modern water ballast tanks. We detect similar levels of genetic subdivision among introduced North American populations and among native European populations. Phylogenetic networks and clustering analyses reveal population structure between sites, a high degree of phylogenetic reticulation within ranges, and phylogenetic splits between European and North American populations. These patterns are inconsistent with phylogeographic structure expected to arise from natural dispersal alone, suggesting human activity eroded ancestral phylogeographic structure between native populations, but was insufficient to overcome divergent processes between naturalized populations and their sources. Our results suggest human activity may alter species' evolutionary trajectories on a broad geographic scale via regional homogenization and global diversification, in some cases precluding historical inference from genetic data.

## Graphical abstract

The same vectors that introduce species to new ranges could move them among native populations, but how human‐mediated dispersal impacts native ranges has been difficult to address because human‐mediated dispersal and natural dispersal can simultaneously shape patterns of gene flow. We investigate genetic patterns from RADseq‐derived SNPs in the amphipod Corophium volutator (*n* = 121; 4,870 SNPs) and the annelid *Hediste diversicolor* (*n* = 78; 3,820 SNPs), which were introduced from Europe to North America, have limited natural dispersal capabilities, are abundant in intertidal sediments, but not commonly found in modern water ballast tanks. Phylogenetic networks and clustering analyses reveal population structure between sites, a high degree of phylogenetic reticulation within ranges, and phylogenetic splits between European and North American populations, which is inconsistent with phylogeographic structure expected to arise from natural dispersal alone and suggests human activity eroded ancestral phylogeographic structure between native populations but was insufficient to overcome divergent processes between naturalized populations and their sources.

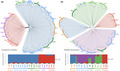

## 1 INTRODUCTION

Humans are moving species beyond the limits of their natural dispersal abilities at unprecedented rates (Seebens et al., 2017). Human‐mediated dispersal erodes biogeographic boundaries (Capinha, Essl, Seebens, Moser, & Pereira, 2015) and is expected to alter the evolutionary trajectories of species associated with human movement through both diversifying and homogenizing effects (Otto, 2018). Human‐mediated dispersal can promote the formation of new allopatric lineages when introduced populations are isolated from their sources (Vellend et al., 2007; e.g., Montesinos, Santiago, & Callaway, 2012), can homogenize genetic diversity (e.g., Alvarez, Hossaert‐McKey, Restoux, Delgado‐Salinas, & Benrey, 2007; Zhan, MacIsaac, & Cristescu, 2010), and dictate patterns of connectivity among introduced populations (Darling, Bagley, Roman, Tepolt, & Geller, 2008; Moran & Alexander, 2014). It is intuitive that the same vectors that facilitate human‐mediated dispersal to and among introduced populations could similarly enable gene flow among native populations (David, 2018; e.g., Carlton & Hodder, 1995).

Whether human‐mediated dispersal alters patterns of gene flow in native ranges is difficult to determine because the contributions of natural and human‐mediated dispersal to gene flow are difficult to disentangle when both processes are ongoing. This is particularly problematic for species whose patterns of colonization and connectivity are understood primarily through genetic data, which is common for many introduced species. For example, the tunicate *Ciona intestinalis* exhibits genetic structure that is not predictable from either geography (as expected from natural dispersal) or shipping networks (as expected from human‐mediated dispersal) among native populations in the English Channel (Hudson, Viard, Roby, & Rius, 2016), with debate over whether populations in North America result from human‐mediated dispersal (Hudson, Johannesson, McQuaid, & Rius, 2019) or natural dispersal (Bouchemousse, Bishop, & Viard, 2016). In many cases, genetic patterns in native ranges are investigated only after the species has been introduced elsewhere, allowing vector activity to potentially influence native populations before they have been observed for the first time. The potential for human‐mediated dispersal to alter patterns of gene flow is therefore likely underestimated, both in terms of the number of species it affects and its magnitude within species.

Here, we investigate how human activity reshapes evolutionary trajectories by exploiting a system of historical changes to shipping technology that resulted in a temporary period of human‐mediated movement of intertidal sediments and the species associated with them. In the 10th Century, the adoption of wooden ships that used sediments shoveled from the intertidal and dredged from near‐shore for ballast enabled long‐distance exchange of abiotic materials and inadvertent hitchhikers in the European Atlantic (Jones, 1976; Stikeman 1832; McGrail, 1989; Ansorge, Frenzel, & Thomas, 2011) and Mediterranean (Casson, 1995). During the 11th‐15th Centuries, the magnitude and reach of these practices increased with the rise of maritime trade around the North and Baltic Seas, with annual rates of sediment relocation in some estuaries on the order of millions of tons (McGrail, 1989; Stikeman, 1832). The exchange of sediments between estuaries via ballast extended in geographic reach during the mid‐15th Century with European exploration and trade in North America. This exchange abruptly declined, and indeed likely ceased entirely, in the late 19th Century when wooden vessels were replaced by steel‐hulled ships that used seawater as ballast (Fofonoff, Ruiz, Steves, & Carlton, 2003). This provides an opportunity to investigate how human‐mediated dispersal impacts native populations: If historical human‐mediated dispersal in the native range was weak, patterns of genetic differentiation and phylogeographic structure among these populations are expected to be more prominent than among populations in the introduced range, whereas homogenization by widespread human‐mediated dispersal is expected to create similar patterns of genetic differentiation and population structure in both the introduced and native ranges.

We focus on two intertidal invertebrates, the amphipod *Corophium volutator* (Pallas, 1766) and annelid *Hediste diversicolor* (Müller, 1776, previously *Nereis diversicolor*), which inhabit soft‐sediment habitat in the Bay of Biscay, North, and Baltic Seas in Europe and the Gulf of Maine and Bay of Fundy in North America. Until recently, they were thought to be native in North America (for review, see Einfeldt & Addison, 2015; Einfeldt, Doucet, & Addison, 2014). Three main lines of evidence suggest they were once moved between Europe and North America in ballast sediments before sediment movement was reduced when water ballast was adopted: (a) these soft‐sediment dwelling intertidal invertebrates are abundant in Europe and North America, but are not reported in intermediate land masses (Iceland and Greenland) which are expected to be inhabited by natural colonizers (Ingolfsson, 1992); (b) genetic diversity in both species is consistent with North American populations representing a subsample from more diverse European populations (Einfeldt & Addison, 2015; Einfeldt et al., 2014); (c) they lack a pelagic phase, brood‐rear their young, and reside in shallow constructed burrows (Peer, Linkletter, & Hicklin, 1986; Scaps, 2002). This is expected to impart limited natural long‐distance dispersal capabilities (via drifting, swimming, or oceanic rafting), as well as a strong association with sediments used in historical ballast, and a weak association with water ballast (Hewitt, Gollasch, & Minchin, 2009). Neither species was found in surveys of modern ballast tanks from 62 ships arriving in North America (Briski, Ghabooli, Bailey, & MacIsaac, 2012), and only a single ship was found to carry any *C. volutator* specimens in a survey of 550 ships in Europe (Gollasch et al., 2002). Prior to their introduction to North America, the same vector that introduced these species from Europe to North America is likely to also have facilitated gene flow among native populations in Europe.

Combined with a restricted natural dispersal ability of both species, geographic barriers are expected to facilitate genetic differentiation between populations reflecting isolation‐by‐distance (IBD; e.g., Bockelmann, Reusch, Bijlsma, & Bakker, 2003; Launey, Ledu, Boudry, Bonhomme, & Naciri‐Graven, 2002), vicariance (e.g., Exadactylos, Geffen, Panagiotaki, & Thorpe, 2003; Panova et al., 2011), and local adaptation (e.g., Butlin et al., 2014). Europe had persistent coastal refugia during the last glacial maximum 18,000 years BP, and both species have distinct mitochondrial lineages consistent with persistence in multiple refugia (Einfeldt & Addison, 2015; Einfeldt et al., 2014; Virgilio, Fauvelot, Costantini, Abbiati, & Backeljau, 2009). Throughout the Holocene, soft‐sediment habitat was fragmented along coastlines by regions of coarse sediment and rocky substrata, with major discontinuities between the British Isles and continental Europe (Andersen et al., 2018). Trace fossils suggest that both species were present on the continental coast of the North Sea and British Isles during this time (Allen & Haslett, 2002; Buller & McManus, 1972; Streif, 1972). In the absence of human‐mediated dispersal, it is therefore expected that population structure (reflected by genetic differentiation) and phylogeographic structure (reflected by geographically structured tree‐like phylogenies) in *C. volutator* and *H. diversicolor* will reflect these physical barriers to movement.

To investigate the net effect of diversifying and homogenizing aspects of human‐mediated dispersal on species' evolutionary trajectories, we compare patterns of genetic divergence using reduced representation genomic sequencing of native European and naturalized North American populations of *C. volutator* and *H. diversicolor*. Depending on different rates of human‐mediated movement, natural dispersal, and natural selection, we could expect three broad patterns of populations genetic structure: (a) if historical human‐mediated movement facilitated range expansion but had little impact on native populations, native populations are expected to exhibit phylogeographic structure and introduced populations are expected to bear high genetic similarity to their sources; (b) if historical human‐mediated movement facilitated gene flow among native populations, it is therefore expected that native populations would exhibit population genetic structure due to contemporary isolation, but not phylogeographic structure due to historical gene flow; and (c) if historical human‐mediated movement facilitated gene flow among native populations, the relative similarity of introduced populations to their sources is expected to decrease due to gene flow between the source and other native populations, thereby increasing measures of between‐range divergence relative to within‐range divergence. Our study provides evidence of the historical impact of human exploration and trade on diversification in introduced invertebrates and draws attention to the potential for human activity to reshape evolutionary trajectories across introduced and native ranges alike.

## 2 METHODS

### 2.1 Sampling and ddRAD‐seq libraries

**FIGURE 1:**
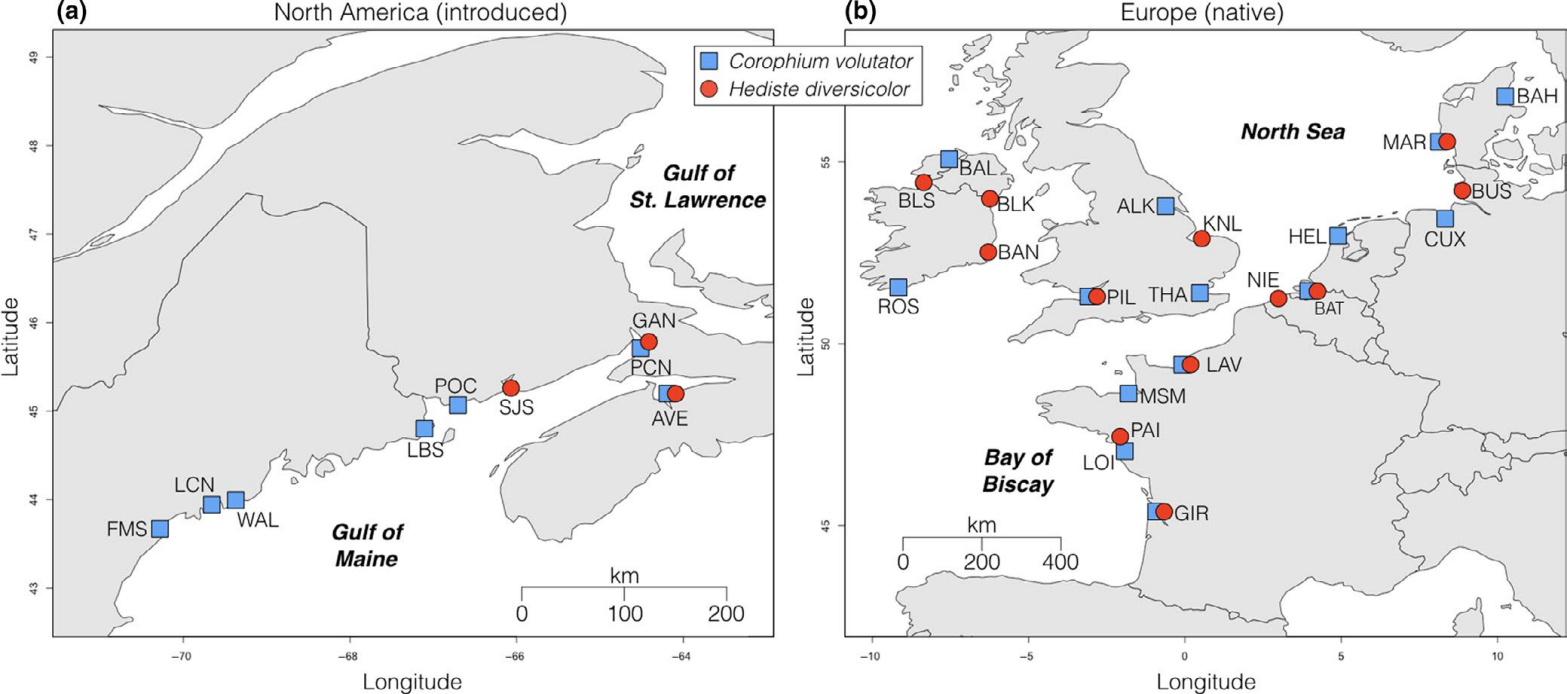
Sample sites. *Corophium volutator* and *Hediste diversicolor* collected from mudflats spanning their known ranges. a, The introduced range in North America. b, The native range in Europe

We sampled 121 individuals of *C. volutator* from 21 sites and 78 individuals of *H. diversicolor* using a trowel and forceps at 15 sites (Table 1; Figure 1) representing the geographic distributions of both species (Einfeldt & Addison, 2015; Einfeldt et al., 2014; Virgilio et al., 2009). In addition to the 78 *H. diversicolor* samples, we identified samples from the Mediterranean (*n* = 4), the North Sea (*n* = 5), and North America outside of the Bay of Fundy (*n* = 17) as cryptic species related to *H. diversicolor* from mitochondrial DNA (Einfeldt et al., 2014; Virgilio et al., 2009). Because we have not sampled the entire native range of these cryptic lineages of the *H. diversicolor* species complex originating in the Mediterranean and Ponto‐Caspian Seas, they were excluded from further analyses. We extensively sampled soft‐sediment habitat along the coast of the Northwest Atlantic from Québec to Massachusetts, and the populations presented here represent the entire present North American latitudinal range of both species. We extracted DNA using QIAGEN DNeasy kits following standard protocols. We prepared individual‐tagged double‐digest restriction‐site‐associated DNA (ddRAD‐seq) (Peterson, Weber, Kay, Fisher, & Hoekstra, 2012) libraries by digesting DNA with Sau96I and MlucI, ligating unique combinations of barcode adapters, combining 25 ng of DNA per individual into libraries, PCR‐amplifying pooled libraries (15 cycles of 98°C for 10 s, 65°C for 30 s, and 72°C for 30 s), and size‐selecting fragments >200 bp by gel extraction and purifying with QIAquick kits following standard protocols. We sequenced samples on five lanes of the Illumina HiSeq platform with 100 bp paired‐end reads at Génome Québec.

**TABLE 1:** Population genetic diversity in *Corophium volutator* and *Hediste diversicolor*

Population	Country	*n*	*H_o_*	*H_e_*	P‐SNPs
*Corophium volutator* (4,870 SNPs, maf > 0.01)					
**Europe**		**79**	**0.1315**	**0.1628**	**2,690**
GIR	France	6	0.1291	0.1295	38
LOI	France	6	0.1241	0.1247	19
MSM	France	6	0.1351	0.1346	25
LAV	France	6	0.1278	0.1303	8
BAT	Netherlands	6	0.1278	0.1306	6
HEL	Germany	5	0.1223	0.1250	6
CUX	Germany	6	0.1300	0.1347	4
MAR	Denmark	6	0.1338	0.1332	10
BAH	Denmark	5	0.1110	0.1140	17
PIL	UK	6	0.1385	0.1379	26
THA	UK	6	0.1474	0.1442	24
ALK	UK	6	0.1438	0.1424	22
ROS	Ireland	6	0.1365	0.1326	50
BAL	Ireland	3	0.1379	0.1238	18
**N. America**		**42**	**0.1106**	**0.1277**	**267**
FMS	USA	6	0.1212	0.1066	0
LCN	USA	6	0.1149	0.1038	0
WAL	USA	6	0.1146	0.1074	0
LBS	USA	6	0.1083	0.1062	5
POC	Canada	6	0.0832	0.0863	8
AVE	Canada	6	0.1190	0.1138	7
PCN	Canada	6	0.1138	0.1120	2
**All**		**121**	**0.1236**	**0.1623**	
*Hediste diversicolor* (3,820 SNPs, maf > 0.01)					
**Europe**		**67**	**0.1404**	**0.1788**	**2,236**
GIR	France	6	0.1679	0.1592	185
PAI	France	6	0.1434	0.1428	88
LAV	France	4	0.1526	0.1468	16
NIE	Belgium	5	0.1373	0.1289	28
BAT	Netherlands	6	0.1486	0.1387	33
BUS	Germany	6	0.1326	0.1291	22
MAR	Denmark	5	0.1367	0.1249	18
PIL	UK	6	0.1401	0.1343	50
KNL	UK	6	0.1349	0.1309	25
BAN	Ireland	5	0.1471	0.1521	17
BLK	Ireland	6	0.1469	0.1357	62
BLS	Ireland	6	0.1222	0.1098	51
**N. America**		**11**	**0.1161**	**0.1235**	**117**
SJS	Canada	5	0.1172	0.1037	31
AVE	Canada	3	0.1231	0.1022	7
GAN	Canada	3	0.1107	0.0925	12
**All**		**78**	**0.1367**	**0.1772**	

Note

Site codes for locations sampled, countries that sites are located in, number of individuals sequenced (*n*), observed heterozygosity (*H_o_*), expected heterozygosity (*H_e_*), and number of SNPs variable in one population but not others (private SNPs). Metrics calculated for Europe, North America, and overall are in bold.

### 2.2 Sequence processing

We demultiplexed raw DNA sequences using *fastq‐multx* v.1.02 (Aronesty, 2013), trimmed barcodes and cut‐site remnants with *fastx_trimmer*, filtered for read quality using *TrimmomaticPE* v.0.33 (Bolger, Lohse, & Usadel, 2014), and merged overlapping paired‐end reads using *PEAR* v.0.9.6 (Zhang, Kobert, Flouri, & Stamatakis, 2013). To maximize the number of nucleotides kept with all sequences a standard length, we trimmed reads to 85 bp. Due to the lack of available genomic resources for *C. volutator* and *H. diversicolor*, to reduce nontarget sequencing we filtered reads for contaminants against standard human and microbial contaminant databases, and custom databases built from publicly available genomes for parasites and algae using Kraken v.1.1.1 (Table S3; Wood & Salzberg, 2014). We assembled a de novo reference and called SNPs using STACKS v.1.35 (Catchen, Hohenlohe, Bassham, Amores, & Cresko, 2013), with forward and reverse reads assembled as different stacks and requiring at least five identical reads for each stack (*m* = 5) to reduce errors introduced through sequencing. We compared results for several combinations of nucleotide mismatches per individual allowed to group stacks (*M* = 2, 4, 6, or 8) and nucleotide mismatches allowed among stacks to form catalog loci (*n* = 2, 4, 6, or 8), which showed a plateau in number of loci retained at *M* = 6 and *n* = 6. To reduce physical linkage, we retained only a single SNP per stack, and to reduce bias from missing data, we retained loci genotyped in 100% of populations (*p* = 1) and at least 50% of individuals per population (*r* = .5) and filtered rare variants by removing SNPs with minor allele frequencies <0.01 (Linck & Battey, 2019).

### 2.3 Genomic diversity and structure

Evolutionary change between introduced populations and their sources can be facilitated by the introduction of genetic diversity from multiple sources (Brawley et al., 2009; Roman & Darling, 2007; Viard, David, & Darling, 2016) and founding effects that facilitate the random or selective establishment of some genotypes over others during the introduction process (Dlugosch & Parker, 2008; Wares, 2005). To assess founding effects associated with introduction, we calculated observed and expected heterozygosity using Adegenet (Jombart & Ahmed, 2011). To investigate the geographic structure of independently evolving lineages, we explored genomic subdivision in *C. volutator* and *H. diversicolor* using ancestry proportion estimates based on sparse non‐negative matrix factorization implemented in the R package SNMF v.1.2 (Frichot, Mathieu, Trouillon, Bouchard, & François, 2014). SNMF estimates ancestry coefficients for individuals from *K* ancestral panmictic gene pools, making no assumptions about the sampled populations that individuals belong to or Hardy–Weinberg equilibrium, and is expected to provide accurate clustering results in the presence of inbreeding and nonequilibrium conditions. We calculated ancestry coefficients for *K* clusters from 1 to the number of sites sampled in five replicate runs. We determined the best number of clusters according to minimum mean cross‐entropy across all runs and calculated admixture proportions for all tested values of *K*. To test for differences in interpopulation genomic differentiation between the native and introduced ranges of *C. volutator* and *H. diversicolor*, we calculated pairwise Reich's *F*_ST_ (Reich, Thangaraj, Patterson, Price, & Singh, 2009), which is unbiased for populations with low sample sizes, among populations in Europe and North America using R code modified from Di Gaetano et al. (2014). To determine the proportion of genetic variance at the levels of individual, population, and continent, we performed AMOVA with ade4 v.1.7 (Dray & Dufour, 2007). To test for correlation between genetic and geographic distance, we regressed Rousset's interindividual genetic distance *â_r_* (Rousset, 2000) against geographic distance with Genepop v.1.1.3 (Rousset, 2008).

### 2.4 Simulated demographic scenarios

To investigate the impact of human‐mediated migration on phylogeographic patterns, we conducted simulations following a complex demographic history with varying degrees of natural and human‐mediated migration, assessing genetic patterns in one of these simulations at different points in time. We simulated SNPs from 10,000 unlinked neutrally evolving diploid loci of 85 base‐pairs each under scenarios of differing magnitudes of human‐mediated migration in continuous time simulations with fastsimcoal2 v.2.6 (Excoffier, Dupanloup, Huerta‐Sánchez, Sousa, & Foll, 2013). Our base model represents the major factors expected to shape phylogeographic structure in this system: population expansion and colonization from a glacial refugia to two island populations and eight mainland populations with stepping‐stone migration, with five introduced populations founded independently from a single mainland population (Figure 3). We used a mutation rate of 2.5 × 10^–8^ per generation per site based on Excoffier et al. (2013), keeping only the first SNP per 85 bp locus for consistency with empirical data and producing allele frequency distributions of SNPs that approximate observed distributions from ddRAD‐seq data. Following expansion, we fixed *N_e_* in each population in order to assess the effects of varying natural and human‐mediated migration on fixation rates, with *N_e_* set to 2,000 for every population except 7 (*N_e_* = 1,000), 15 (*N_e_* = 1,000), and 16 (*N_e_* = 200). Reduced values of *N_e_* were included to visualize the effect of increased drift. We used two generations per year, reflecting reproductive cycles of *C. volutator* and *H. diversicolor* (Dales, 1950; Peer et al., 1986). We set the magnitude of within‐range human‐mediated migration (*M_H_*) active in this system over 1,800 generations (corresponding to the ~900‐year window of widespread ballast sediment exchange) to be an order of magnitude less than, equal to, and an order of magnitude more than the magnitude of natural migration (*M_N_*), followed by a return to natural migration matrices for 200 generations (~100 years).

### 2.5 Demographic complexity and phylogenetic conflict

Species with limited capacities for natural dispersal are expected to exhibit tree‐like phylogenetic patterns reflecting demographic history and geographic barriers to gene flow, while gene flow enabled by human‐mediated dispersal is expected to decrease the phylogenetic concordance across loci, creating phylogenetic conflict reflected by low bootstrap support for nodes and high measures of reticulation. To examine phylogenetic relationships in *C. volutator, H. diversicolor,* and simulated datasets, we created unrooted networks based on uncorrelated *p*‐distances between individuals using a neighbor‐net method in Splitstree v.4.13 with 1,000 bootstrap iterations (Huson & Bryant, 2005). To compare levels of phylogenetic conflict in empirical and simulated data, we quantified network reticulation by calculating the mean Delta scores across networks (Holland, Huber, Dress, & Moulton, 2002). High levels of Delta suggest phylogenetic conflict. Delta is a ratio of path lengths between quartets of populations, for which a value of 0 corresponds to low phylogenetic conflict and a completely tree‐like phylogeny, and a value of 1 corresponds to equal distances between all populations in the quartet.

### 2.6 Scans for selection

We performed scans for positive selection using two methods that are reported to perform well in demographic scenarios involving hierarchical spatial structure and population expansion: an *F*_ST_ based approach implemented in OutFLANK v.0.2 (Whitlock & Lotterhos, 2015) and a principle components based approach implemented in PCAdapt v 4.1 (Luu, Bazin, & Blum, 2017). To correct for bias arising from multiple tests, we calculated *q*‐values from *p*‐values produced by each test with a false discovery rate threshold of 5%. For PCAdapt, we chose the optimal number of genetic components *K* based on Cattell's rule applied to scree plots, which was *K* = 3 for both species. To assess false‐positive ratios, we performed both scans for selection on SNPs from data simulated using a strictly neutral model of evolution.

## 3 RESULTS

### 3.1 Samples and SNPs

We genotyped 121 *C. volutator* and 78 *H. diversicolor* individuals from mudflats covering the geographic extent of their distributions, with each sample site representing a population unit (Figure 1). Sequencing 2 × 100 base‐pair paired‐end reads of double‐digest restriction‐site‐associated DNA at >10× average coverage and keeping the first single nucleotide variation (SNP) per locus produced a total of 4,870 SNPs for *C. volutator* and 3,820 SNPs for *H. diversicolor* (Table S4).

### 3.2 Reduced genetic diversity in North American populations

The number of SNPs polymorphic in one continent but not the other (corrected for the number of individuals) was 6.4 in North America and 34.1 in Europe for *C. volutator*, and 10.6 in North America and 33.4 in Europe for *H. diversicolor*. The average number of SNPs polymorphic in one population but not others (corrected for the number of individuals) was 0.5 (*SD* = 0.6) in North America and 3.5 (*SD* = 2.3) in Europe for *C. volutator*, and 4.2 (*SD* = 1.9) in North America and 8.6 (*SD* = 7.8) in Europe for *H. diversicolor* (Table 1). Average observed heterozygosity in North American populations was 14.9% lower for *C. volutator* and 16.6% lower for *H. diversicolor* than in European populations (Table 1). This is similar to the 18.7% average difference between ranges observed across a review of 70 recently introduced species (Dlugosch & Parker, 2008).

### 3.3 Genetic divergence between North America and Europe

**FIGURE 2:**
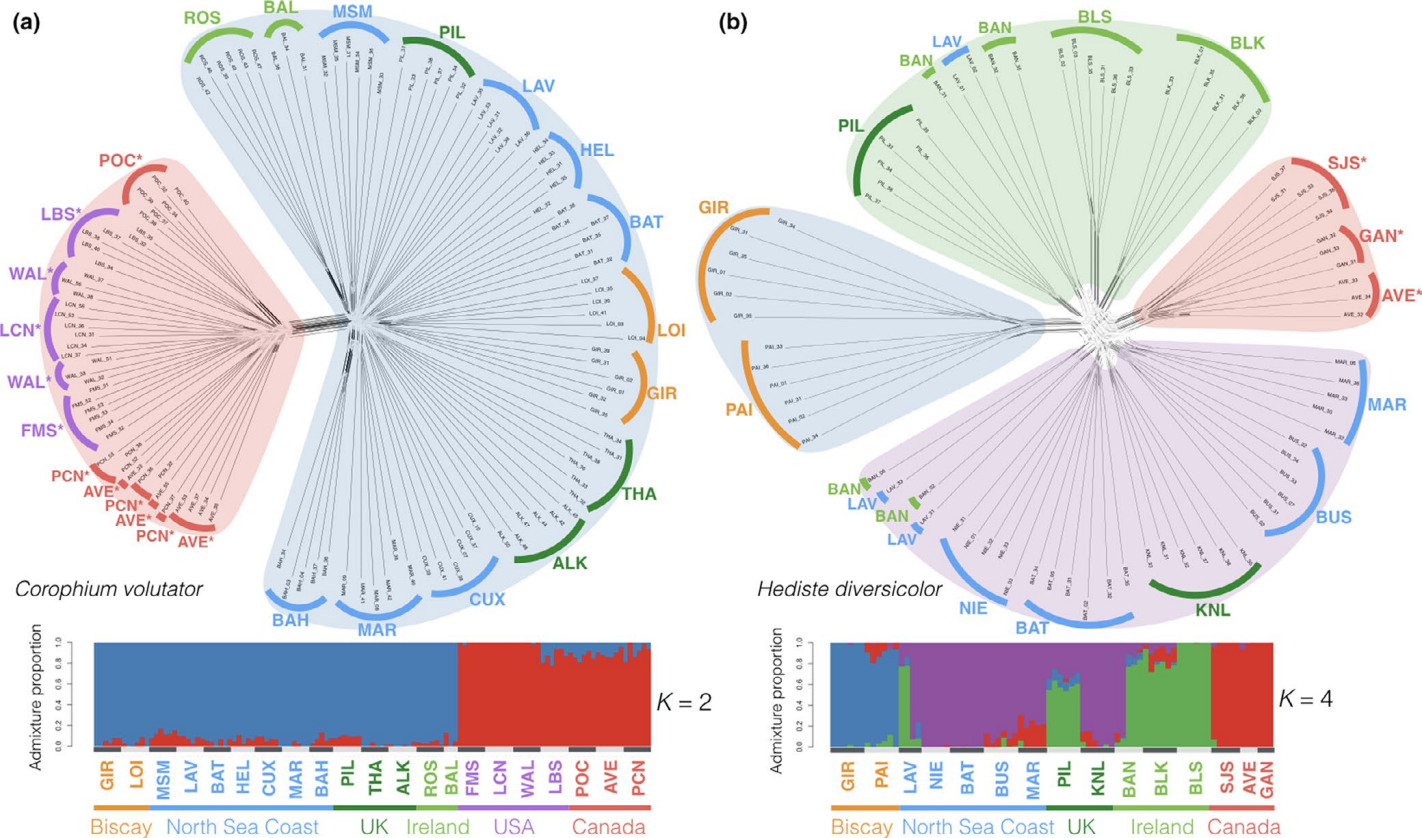
Population genetic structure. a‐b, Neighbor‐joining networks for *Corophium volutator* (4,870 SNPs) and *Hediste diversicolor* (3,820 SNPs) show clustering of individuals by sampling site, division between native versus introduced ranges, and reticulation between populations within each range. Lighter network shading indicates weaker bootstrap support due to phylogenetic conflict. * Introduced samples. c and d, Admixture proportions for individuals in *K* optimal genotypic clusters with SNMF

Consistent with the scenario that introduced populations are isolated from and are evolving independently of their native ranges, neighbor‐joining phylogenetic networks show strong support for separation between the introduced and native ranges of both species (Figure 2). Pairwise genetic differentiation calculated using Reich's *F*_ST_ supports strong genetic structuring between North American and European populations for *C. volutator* (*F*_ST‐NA‐EU_ = 0.237; *SD* = 0.036) and *H. diversicolor* (*F*_ST‐NA‐EU_ = 0.244; *SD* = 0.053). Calculation of *F*_ST_ on simulated data shows no bias with low sample sizes (Table S1). The proportion of genetic variation between continents was greater than the proportion of variation between populations within continents for *C. volutator* (14.7% between continents, 10.1% between populations within continents), but not for *H. diversicolor* (8.8% between continents, 17.5% between populations within continents). The proportion of variation between individuals within populations was relatively low for both *C. volutator* (5.2%) and *H. diversicolor* (2.3%). In *C. volutator,* the best‐fit number of genetic clusters (*K* = 2) differentiates North American from European individuals, and the best‐fit value for each continent separately is *K* = 1 for North America and *K* = 2 for Europe, with two populations from Ireland (ROS & BAL) and one from Denmark (BAH) forming a separate cluster (consistent with results for Figure S1 *K* = 3). Each of these clusters analyzed separately has a best‐fit value of *K* = 1. In *H. diversicolor,* the best‐fit number of clusters (*K* = 4) differentiates North American individuals from three genetic clusters in Europe, and analyses of these clusters separately have a best‐fit value of *K* = 1. Increasing *K* beyond its optimal value reveals increasingly fine‐scale clustering that corresponds to geography in both species, particularly among native populations, but does not group North American individuals with European clusters (Figure S1).

### 3.4 Genetic structure among European populations

Phylogenetic networks and genetic clustering recover nearly all population groups with high fidelity (Figure 2, Figure S1), consistent with a scenario of limited natural dispersal and fragmented habitat. Limited dispersal capability is expected to facilitate higher gene flow between nearby population pairs than across more distant pairs, leading to patterns of IBD (Bradbury, Laurel, Snelgrove, Bentzen, & Campana, 2008; Jenkins et al., 2010; Kelly & Palumbi, 2010). Consistent with this expectation, genetic differentiation among European samples exhibits IBD for both species (Figure S2), with a less positive and correlated relationship for *C. volutator* (1.009e−04 *â_r_*/km; adjusted *r* ^2^ = .299; *p* < .001) than for *H. diversicolor* (2.473e−04 *â_r_*/km; adjusted *r* ^2^ = .431; *p* < .001). IBD in the native range was less positive and correlated than in the introduced range for *C. volutator* (1.738e−04 *â_r_*/km; adjusted *r* ^2^ = .123; *p* < .001) and *H. diversicolor* (1.232e−03 *â_r_*/km; adjusted *r* ^2^ = .783; *p* < .001). Pairwise estimates of *F*_ST_ among European populations was similar to that observed among North American populations for *C. volutator* (*F*_ST‐NA_ = 0.116; *F*_ST‐EU_ = 0.109; *p* = .492) and for *H. diversicolor* (*F*_ST‐NA_ = 0.121; *F*_ST‐EU_ = 0.170; *p* = .091).

### 3.5 Phylogenetic conflict between European populations

**FIGURE 3:**
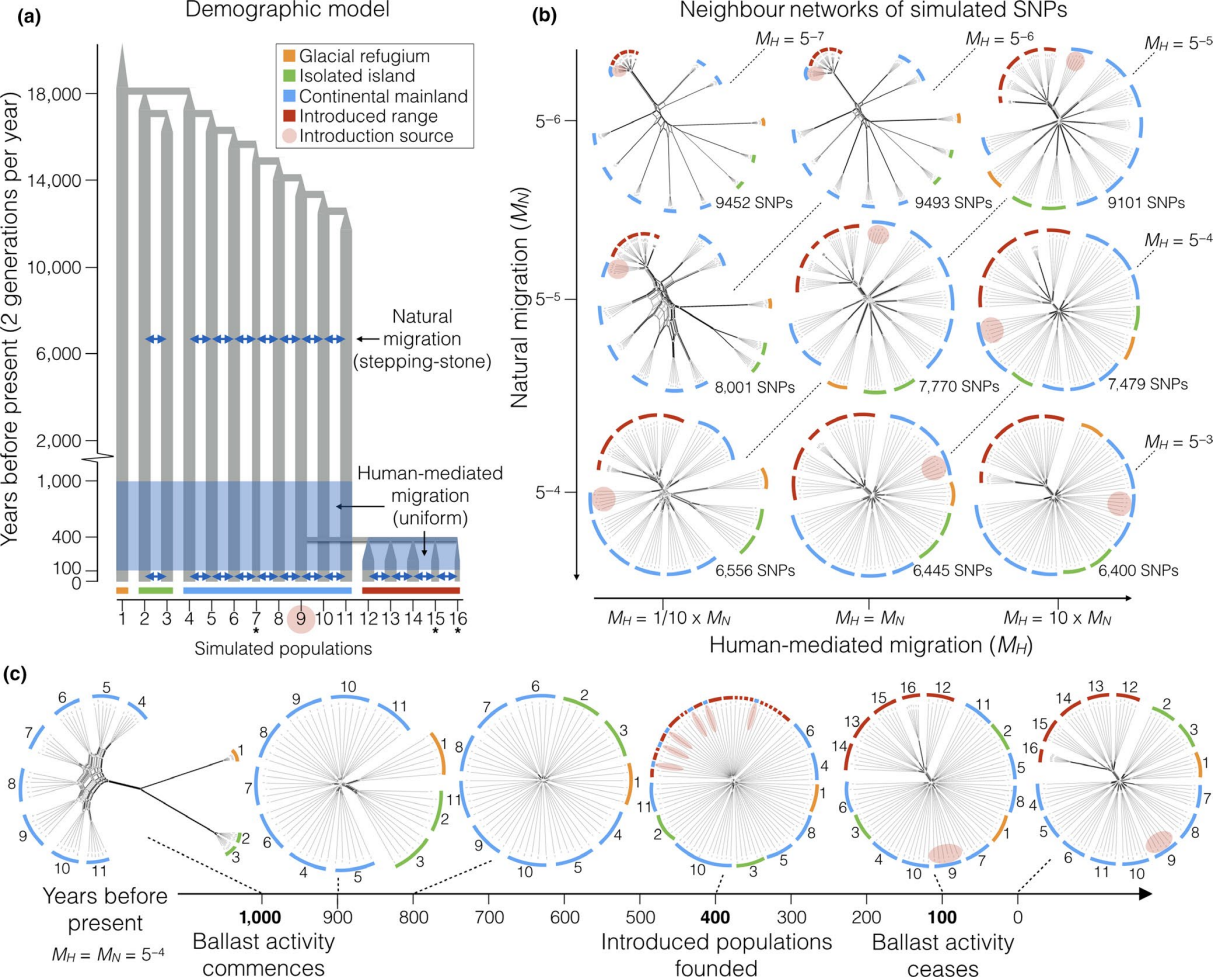
Human‐mediated migration erases ancestral phylogeographic structure. a, Demographic model for simulated genetic data: population expansion, post‐Pleistocene colonization from a glacial refugium, natural migration (*M_N_*) between adjacent populations in discontinuous habitat, founding effects during introduction, and temporary human‐mediated migration (*M_H_*) based on two generations per year. * *N_e_* reduced by 50% in populations 7 and 15, by 90% in population 16 relative to majority of populations to visualize the effects of increased drift. b, Neighbor networks of SNPs from simulated genetic data for different values of *M_N_* and *M_H_*, with the lowest values in the top left representing a scenario with the least amount of dispersal. c, Time series of a single simulation (*M_N_* = 5^–4^, *M_H_* = 5^–4^) shows initial phylogeographic structure, erosion of branches after the onset of human‐mediated dispersal, and early stages of phylogeographic structure 100 years after human‐mediated dispersal has ceased

Long‐standing isolation between continental Europe and the British Isles and stepping‐stone migration between populations distributed along >2,500 km of European coast is expected to produce branching networks that reflect colonization history, geographic location, and major barriers to dispersal (Figure 3; and see Macher et al., 2015). Contrary to the pattern of phylogeographic structure expected for this scenario, populations from the British Isles did not form monophyletic groups for *C. volutator* or *H. diversicolor*, and populations from the European continental coast did not cluster strictly according to geographic location (Figure 2). Empirical values of mean phylogenetic reticulation are high for both *C. volutator* and *H. diversicolor* (Delta = 0.030 for both species), with similar values for simulated data when human‐mediated migration was high (Table 2). Compared to genetic differentiation between ranges, phylogenetic networks show little divergence in the European range of *C. volutator* and comparable divergence for only the two Bay of Biscay populations in *H. diversicolor*. Both species exhibit relatively short branch lengths and low bootstrap support for native interpopulation connections. While individuals sampled from the same site generally cluster together, networks show extensive reticulation indicating nearly equal phylogenetic conflict between all pairs of native populations, with two notable exceptions for both of *C. volutator* and *H. diversicolor*. In *C. volutator*, the two most northern populations in Denmark and the two populations from Ireland each form clusters. In *H. diversicolor*, the two most southern populations from the Bay of Biscay form a cluster that is more separated from the network than any other group of individuals, and individuals from one population in Ireland (BAN) and one population in France (LAV) are interspersed in two different clusters.

**TABLE 2:** Phylogenetic conflict in *Corophium volutator*, *Hediste diversicolor*, and simulated data

Data type	Dataset	*M_N_*	*M_H_*	Delta
Empirical	*Corophium volutator*			0.30
	*Hediste diversicolor*			0.30
Simulated	A	5^–6^	5^–7^	0.12
	B	5^–6^	5^–6^	0.14
	C	5^–6^	5^–5^	0.21
	D	5^–5^	5^–6^	0.15
	E	5^–5^	5^–5^	0.19
	F	5^–5^	5^–4^	0.29
	G	5^–4^	5^–5^	0.20
	H	5^–4^	5^–4^	0.29
	I	5^–4^	5^–3^	0.30
Simulated (time series)	1,000 YA	5^–4^	0 to 5^–4^	0.18
	900 YA	5^–4^	5^–4^	0.31
	800 YA	5^–4^	5^–4^	0.40
	400 YA	5^–4^	5^–4^	0.41
	100 YA	5^–4^	5^–4^ to 0	0.33
	Present	5^–4^	0	0.29

Note

Degree of reticulation in phylogenetic networks (Delta) resulting from conflicting gene trees among loci, strength of natural (*M_N_*) and human‐mediated (*M_H_*) migration vectors for end‐point and time series simulations. Migration values with two values represent sampling at transition point between migration matrices (Figure 3a). High levels of Delta suggest phylogenetic conflict.

### 3.6 Human‐mediated gene flow erodes ancestral phylogeographic structure

To investigate how different scenarios of human‐mediated and natural dispersal influence phylogeographic and population genetic structure, we simulated neutrally evolving populations with two generations per year undergoing postglacial expansion and colonization, isolation between three regions in the native range, introduction to a new range (400 years ago), and varying strengths of natural (stepping‐stone) and human‐mediated (active across all populations within each range) gene flow at times corresponding to ballast sediment use in Europe (100–1,000 years ago). The effects of human‐mediated gene flow depend on the magnitude of natural gene flow between populations, but phylogenetic networks of simulated data show two emergent patterns (Figure 3): (a) increasing human‐mediated gene flow causes relationships between native populations to become less tree‐like and more reticulated (Table 2); (b) increasing human‐mediated gene flow changes introduced populations from clustering with their ancestral source to being their own divergent cluster. Genotypes sampled at different time points from a single simulation prior to the onset of human‐mediated dispersal produce networks that recover phylogeographic structure (Figure 3c), with isolated populations having longer branch lengths and continental mainland populations having positions reflecting their geographic location. After the initiation of human‐mediated migration, Delta increases from 0.18 to 0.40 over 200 years (Table 2), indicating a rapid increase of phylogenetic conflict among native populations. When new populations are founded, they cluster with their source, but this is eroded as human‐mediated gene flow continues between native populations. Over the 300 years from the establishment of introduced populations to the time when human‐mediated gene flow ceases, Delta decreases from 0.41 to 0.33, reflecting a reduction in mean phylogenetic conflict due to limited gene flow between ranges. From the time that human‐mediated gene flow ceases to one hundred years later, branch lengths elongate, populations from the continental mainland begin to re‐order into positions corresponding to their geographic location, and Delta further decreases to 0.29, reflecting a further decrease in mean phylogenetic conflict.

### 3.7 High false‐positive rates in scans for selection

The erosion of ancestral phylogeographic structure precludes accurate inference of premixture demographic history using bi‐allelic SNP data (Appendix S1), but whether it violates the assumptions of selection tests relying on SNP frequencies is less obvious. To determine whether selection can be inferred from distribution‐wide patterns of genetic diversity in this system, we performed scans for positive selection on *C. volutator*, *H. diversicolor,* and data from simulations using two methods reported to perform well regardless of demographic history: OutFLANK (Whitlock & Lotterhos, 2015), which infers the neutral distribution of *F*_ST_, and the PCA‐based PCAdapt (Luu et al., 2017). OutFLANK did not detect outlier SNPs in either species or the simulated data sets. PCAdapt identified a large proportion of SNPs as outliers in *C. volutator* (16% of SNPs) and *H. diversicolor* (23% of SNPs), but these results were well within the 13%–46% (mean *α* = .27, *SD* = 0.11) range of false‐positive ratios identified in simulated data (Table S2). These results suggest that the demographic histories of these species shifted allele frequencies strongly enough to mask differences resulting from directional selection even if it is present, precluding accurate inference of selection using frequency‐based scans. Whether selection contributes to the patterns of genetic divergence observed from genome‐wide SNPs in *C. volutator* and *H. diversicolor* thus remains uncertain.

## 4 DISCUSSION

We exploited historical introductions of two intertidal invertebrates with limited natural capabilities for dispersal to determine the effects of human activity on their evolutionary trajectories. North American populations had lower metrics of genetic diversity than European populations, consistent with both species having undergone founder effects or genetic bottlenecks as a result of introduction from Europe, which may contribute to divergence between the introduced and native ranges. Native populations are expected to exhibit phylogeographic structure reflecting landscape features and demographic history, and if structure is present in a native range then introduced populations are expected to be more genetically similar to their sources than other native populations (Geller, Darling, & Carlton, 2010). However, we found genetic divergence between the introduced and native ranges of both species and extensive phylogenetic conflict among populations within either range. These results support the hypotheses that (a) human‐mediated dispersal can facilitate establishment of introduced populations that follow evolutionary trajectories that are independent from their native sources if human activity ceases; and (b) human activity can overcome natural barriers to dispersal between native populations and erode ancestral phylogeographic structure.

We detected evidence of contemporary gene flow in *C. volutator* or *H. diversicolor* between populations in adjacent mudflats in a limited number of cases, suggesting that rates of natural dispersal vary by location due to interactions with environmental features such as regional currents. Natural dispersal of these species over short distances within either continental coastline is likely facilitated by brief bouts of swimming associated with incoming tides (Aberson, Bolam, & Hughes, 2011; Drolet & Barbeau, 2009) and by rafting of frozen sediments (MacFarlane, Drolet, Barbeau, Hamilton, & Ollerhead, 2013) in North America where winter temperatures are more severe than in Europe. For *C. volutator*, only two pairs of adjacent populations were not distinct in phylogenetic networks or clustering analyses, suggesting gene flow between them. The first pair of populations are separated by ~40 km in the Gulf of Maine, and the second pair are separated by ~130 km in the inner Bay of Fundy, which has extremely high tidal velocities. The proximity of these populations suggests they are connected by natural dispersal. In *H. diversicolor*, individuals from two populations separated by ~690 km, in Wexford, Ireland (Bannow Bay; BAN) and Normandy, France (Seine estuary; LAV), form two distinct genetic groups with individuals from each population in both clusters (Figure 2). This suggests either reciprocal exchange between these sites or movement from two other unsampled populations to both of these sites. However, DNA from individuals that do not group with the expected genetic clusters (LAV01, LAV02, BAN02, and BAN06) was adjacent on plates during library preparation, and mislabeling cannot be ruled out as a potential cause of this pattern. If these two estuaries are connected, the likely recent connectivity between them appears to be a rare exception to the general pattern of contemporary isolation between sites. While modern ballast water transport is a contemporary dispersal vector for other crustaceans and annelids (Briski et al., 2012), the limited evidence of contemporary connectivity between distant populations of *C. volutator* and *H. diversicolor* supports previous biogeographic and genetic evidence that historical dispersal via ballast sediments was the primary vector of introduction for these two species.

Phylogenetic networks and genetic clustering of individuals are consistent with the hypothesis that introduced populations of *C. volutator* and *H. diversicolor* are isolated from, and are evolving independently of, their native ranges. Multiple sources, introduction from a single admixed native population, postintroduction genetic change in source populations via gene flow with other native populations, or a combination of these processes may have influenced the evolution of *C. volutator* and *H. diversicolor*. We found that introduced populations did not cluster with any populations in the native range of both species, impeding inference of historical colonization routes (further discussed in Appendix S1). This contrasts with genetic patterns from mitochondrial DNA, which found North American haplotypes to be subsampled from those found in European populations with no discernible divergence (Einfeldt & Addison, 2015; Einfeldt et al., 2014). Noncongruent patterns between mitochondrial and genomic DNA are expected due to recombination between nuclear markers but not mitochondrial sequences, consistent with patterns seen in more recently introduced species (Ayari et al., 2017; Jeffery et al., 2017). Divergence between Europe and North America may be in part due to rapid evolution of introduced populations, which can arise from neutral (e.g., Baker & Moeed, 1987; Shultz, Baker, Hill, Nolan, & Edwards, 2016) and selective (e.g., Colautti & Barrett, 2013; Huey, Gilchrist, Carlson, Berrigan, & Serra, 2000) processes and ultimately lead to reproductive isolation between introduced populations and their sources (e.g., Montesinos et al., 2012). We detected few variants unique to the introduced range of either species (Table 1), suggesting little influence of mutation after introduction. Variation in number of private SNPs per population corrected for number of individuals was high, which could result from different effective population sizes, adaptation, demographic history, or patterns of connectivity particular to each population. While the observed patterns do not rule out the possibility that genetic material may have been contributed from multiple divergent sources, which could rapidly lead to genetic divergence (Geller et al., 2010), this is not sufficient to explain phylogenetic conflict between European populations.

Although most populations of *C. volutator* and *H. diversicolor* are genetically distinct, genetic patterns suggest that historical human activity likely altered their evolutionary trajectories. Native ranges are expected to have high levels of genetic divergence among geographically isolated populations—constituting phylogeographic structure—particularly in species with poor natural dispersal capabilities and fragmented habitat (Bilton, Paula, & Bishop, 2002; Bohonak, 1999). Populations evolving in isolation are expected to diverge in monophyly, with IBD resulting in phylogenetic patterns that reflect geography, while gene flow between populations is expected to cause conflicting phylogeographic signals between genomic regions (Avise, 2000; Maggs et al., 2008). We expected greater pairwise genetic differentiation and stronger patterns of IBD in Europe than in North America due to greater geographic separation and longer time since becoming naturalized. IBD was evident in Europe and North America for both *C. volutator* and *H. diversicolor*, but counter to our predictions it was stronger in North America, suggesting that the factors restricting movement differ between ranges or over different geographic scales. Long‐standing IBD resulting from natural dispersal between adjacent populations is expected to produce phylogenetic networks with topologies that reflect geography (e.g., Jeffries et al., 2016), and limited dispersal between mainland and island habitats is expected to produce branching networks (e.g., first network in Figure 3c). Contrary to these expectations, *C. volutator* and *H. diversicolor* had reticulation between populations. While bi‐allelic markers do not provide information about the timing and magnitude of pulses of gene flow over short evolutionary timescales, the restricted natural dispersal capabilities of *C. volutator* and *H. diversicolor*, rare evidence of contemporary long‐distance connectivity, phylogenetic reticulation, and discord between genetic structure and geography are consistent with historical human‐mediated dispersal among estuarine habitats. Human transport can promote human‐mediated connectivity among introduced populations (Lacoursière‐Roussel et al., 2012; Reem, Mohanty, Katzir, & Rinkevich, 2013; Voisin, Engel, & Viard, 2005), and our results suggest that human endeavor may also facilitate connectivity among native populations.

The potential for human activity to facilitate genetic admixture between native populations may have both undesirable and desirable consequences for conservation. Human‐mediated dispersal may increase the risk of species to become invasive beyond their natural distributions by facilitating secondary contact among native populations and thereby increasing their adaptive potential (e.g., Anderson et al., 2018; Bertelsmeier et al., 2018; Lehnert et al., 2018; Vandepitte et al., 2014). We provide the first empirical evidence that human‐mediated dispersal may decrease our ability to interpret history from genetic data in native as well as introduced ranges by obscuring ancestral phylogeographic structure arising from natural processes, erasing the signatures of demographic history recorded in DNA (Crozier & Schulte‐Hostedde, 2015). On the other side of the same coin, human‐mediated gene flow could enhance the introduction of advantageous alleles to populations that are not otherwise able to respond to anthropogenic pressures such as climate change, akin to directed efforts of assisted gene flow (Aitken & Bemmels, 2016).

The effect of human‐mediated movement on patterns of gene flow within native ranges is likely underestimated. The native ranges of other introduced marine species often exhibit unexpected genetic patterns, which could potentially be explained by human patterns of human movement. For example, in the invasive green crab *Carcinus maenas* mitochondrial haplotypes typically found in the South of Spain have also been found in the UK (Darling et al., 2008), and an Atlantic lineage of the marine gastropod *Stramonita haemastoma* may potentially have been cryptically introduced to the Mediterranean, where it hybridizes with a Mediterranean lineage (Ayari et al., 2017). The degree of association with contemporary vectors of transport is unknown for many taxa, due to limited availability of vessel surveys and the challenges of correctly identifying small inconspicuous species across all their life stages (Haydar, 2012). Additional genetic analyses of historically introduced species could help further our understanding of how frequently each of these unintentional outcomes has occurred (Rius & Darling, 2014), providing insight into expected evolutionary outcomes of contemporary human activity.

## CONFLICT OF INTEREST

The authors declare that they have no potential sources of conflict of interest.

## AUTHOR CONTRIBUTIONS

**Anthony L. Einfeldt:** Conceptualization (lead); data curation (lead); formal analysis (lead); funding acquisition (supporting); investigation (lead); methodology (lead); project administration (equal); visualization (lead); writing – original draft (lead); writing – review & editing (lead). **Linley K. Jesson:** Conceptualization (supporting); supervision (equal); writing – review & editing (supporting). **Jason A. Addison:** Conceptualization (supporting); funding acquisition (lead); project administration (equal); supervision (equal); writing – review & editing (supporting).

## Supporting information

Appendix S1

## Data Availability

SNP datasets, high resolution versions of images, and code for processing ddRAD‐seq libraries and calculating Reich's *F*_ST_ estimator is available on github (https://github.com/einfeldt/ddrad_process) and Dryad (LINK PROVIDED DURING SUBMISSION). Raw data are available from NCBI's SRA under BioProject ID: PRJNA577895; accessions SAMN13041043:SAMN13041241.
