# Supplementary material for: Historical human activities reshape evolutionary trajectories across both native and introduced ranges
Source: Ecol Evol. 2020 May 24;10(13):6579–92. doi: 10.1002/ece3.6391 (PMC7381589; doi:10.1002/ece3.6391)
Supplement: Supplementary file 1 — Appendix S1 [file ECE3-10-6579-s001.docx]

**
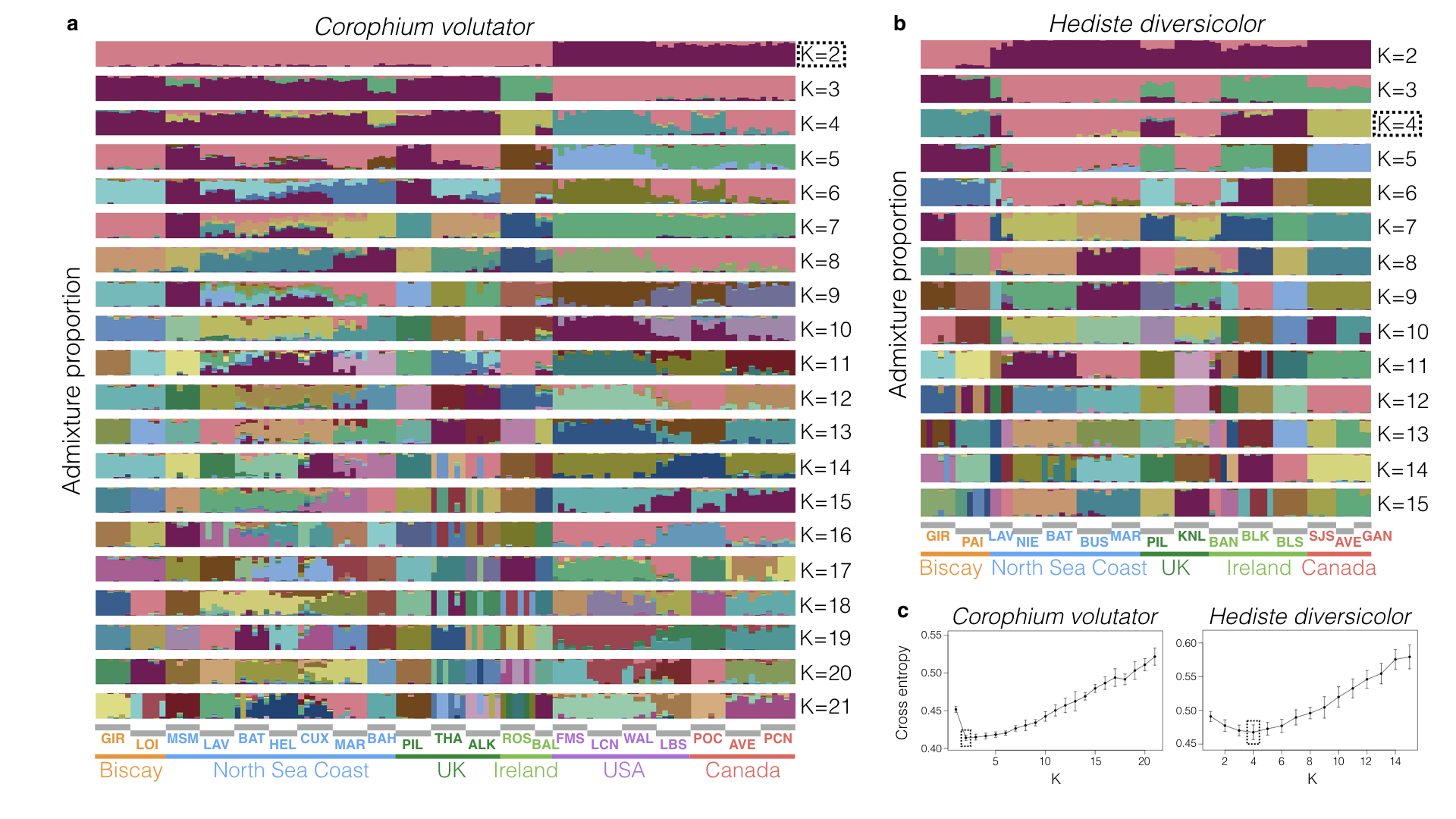
**

**Figure S1 | Hierarchical genetic structure. a-b,** Assignment proportions for individuals to *K* genotypic clusters (separate colours for each independent run of *K*) according to minimum cross-entropy computed with SNMF show genetic subdivision at increasingly fine geographic scales as *K* is increased.

**Figure S2 | Isolation by distance.** Linear models of Rousset’s inter-individual genetic distance (*â_r_*) against geographic distance (km) for *Corophium volutator* (**a-b**) and *Hediste diversicolor* (**c-d**).

**d**

**b**

**a**

**
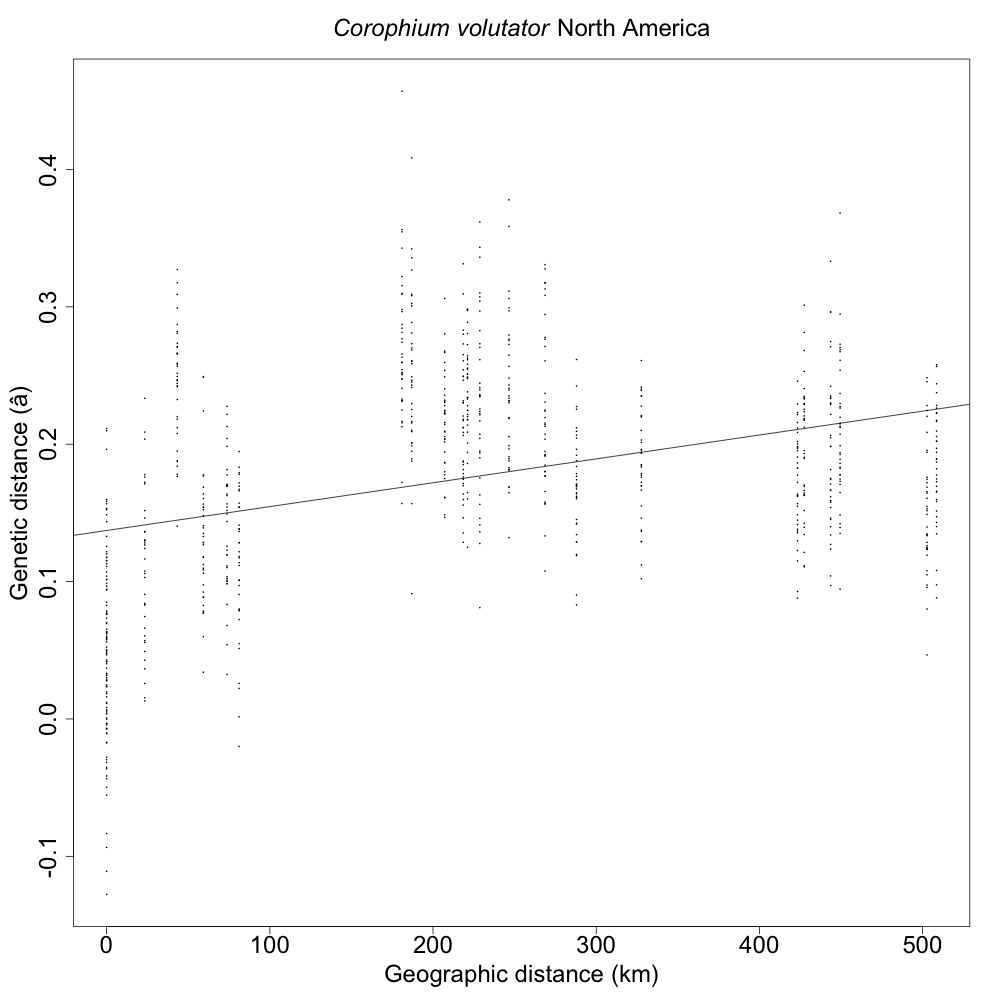

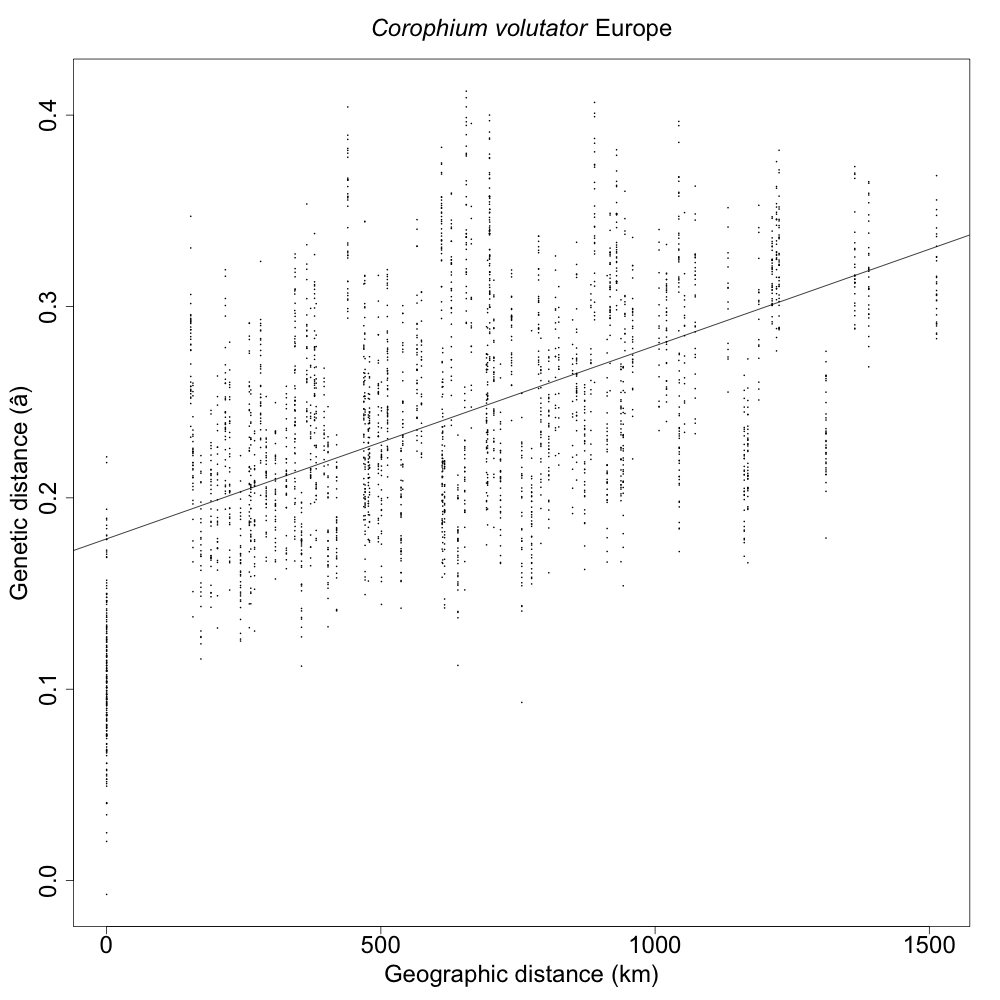
**

**c**

**
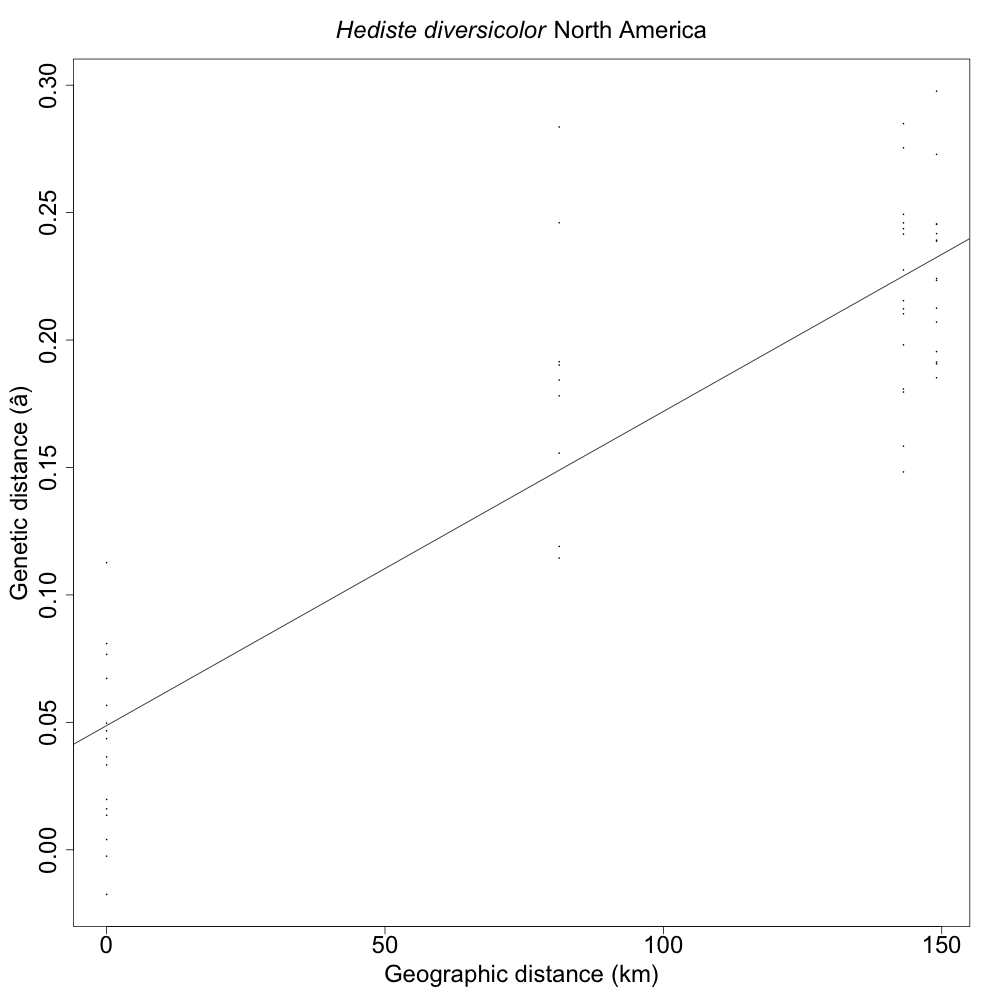

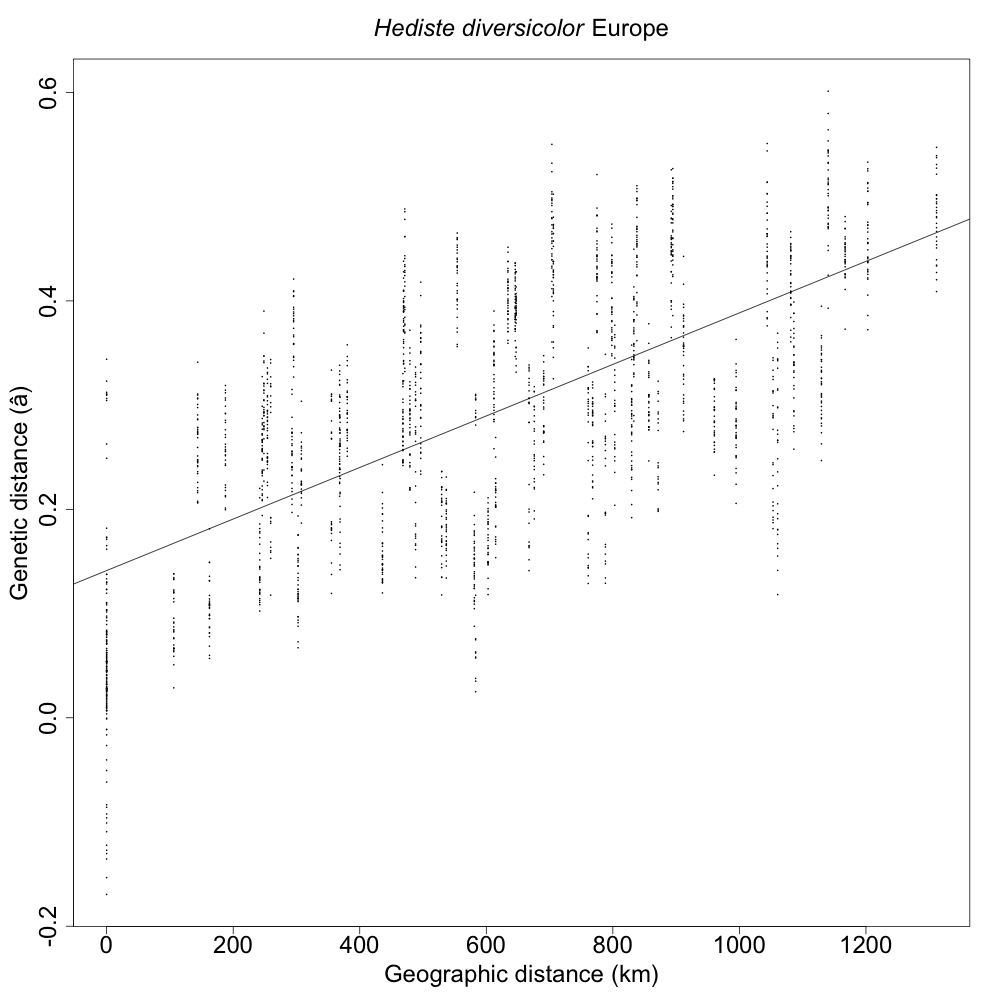
**

**Table S1 | Mean pairwise population comparisons of Reich’s F_ST_ and standard deviations for simulated datasets with different sample sizes*.***

| **n** | **Mean F_ST_** | **SD F_ST_** |
| --- | --- | --- |
| 2 | 0.2575 | 0.0492 |
| 3 | 0.2538 | 0.0460 |
| 6 | 0.2564 | 0.0496 |
| 12 | 0.2586 | 0.0470 |
| 24 | 0.2591 | 0.0486 |

**Table S2 | False positive ratios of selection scans on data simulated with a strictly neutral evolutionary model.** Natural (*N_m_*) and human-mediated (*H_m_*) migration rates, number of SNPs detected in 10,000 simulated loci of 85 bp each, number of outliers detected, and false positive ratios of outlier detection ( = number of outlier detected / number of SNPs) using PCAdapt.

| Simulated data set | Natural migration *N_M_* | Human-mediated migration *H_M_* | Number of SNPs | Number of outliers detected | False positive ratio |
| --- | --- | --- | --- | --- | --- |
| i | 5E-06 | 5E-07 | 9452 | 4326 | 0.4577 |
| ii | 5E-06 | 5E-06 | 9493 | 3747 | 0.3947 |
| iii | 5E-06 | 5E-05 | 9101 | 1954 | 0.2147 |
| iv | 5E-05 | 5E-06 | 8001 | 2561 | 0.3201 |
| v | 5E-05 | 5E-05 | 7770 | 1502 | 0.1933 |
| vi | 5E-05 | 5E-04 | 7479 | 2355 | 0.3149 |
| vii | 5E-04 | 5E-05 | 6556 | 858 | 0.1309 |
| viii | 5E-04 | 5E-04 | 6445 | 1553 | 0.2410 |
| ix | 5E-04 | 5E-03 | 6400 | 1248 | 0.1950 |

**Table S3 | Parasite and algal genomes filtered from genomic sequence data.** Species grouped by databases constructed with Kraken, genome size, assembly used, and taxonomic identification code.

| **Organism** | **Size (MB)** | **genomeaccession_keys** |
| --- | --- | --- |
| Trematodes | 7066 |  |
| *Clonorchis sinensis* | 547 | GCA_000236345 |
| *Fasciola hepatica* | 1275 | GCA_000947175 |
| *Opisthorchis viverrini* | 620 | GCF_000715545 |
| *Schistosoma haematobium* | 376 | GCF_000699445 |
| *Schistosoma japonicum* | 403 | GCA_000151775 |
| *Schistosoma mansoni* | 365 | GCA_000237925 |
| *Dicrocoelium dendriticum* | 548 | GCA_000950715 |
| *Echinostoma caproni* | 835 | GCA_000950555 |
| *Schistosoma curassoni* | 344 | GCA_000951415 |
| *Schistosoma margrebowiei* | 367 | GCA_000951435 |
| *Schistosoma mattheei* | 341 | GCA_000951455 |
| *Schistosoma rodhaini* | 343 | GCA_000951475 |
| *Trichobilharzia regenti* | 702 | GCA_000950905 |
| Nematodes | 6855 |  |
| *Ancylostoma ceylanicum* | 313 | GCA_000402015 |
| *Ancylostoma duodenale* | 333 | GCA_000816745 |
| *Angiostrongylus cantonensis* | 6 | GCA_000950995 |
| *Ascaris suum* | 263 | GCA_000187025 |
| *Brugia malayi* | 94 | GCF_000002995 |
| *Brugia pahangi* | 84 | GCA_001280985 |
| *Bursaphelenchus xylophilus* | 73 | GCA_000231135 |
| *Caenorhabditis angaria* | 80 | GCA_000165025 |
| *Caenorhabditis brenneri* | 190 | GCA_000143925 |
| *Caenorhabditis briggsae* | 108 | GCF_000004555 |
| *Caenorhabditis elegans* | 100 | GCF_000002985 |
| *Caenorhabditis japonica* | 166 | GCA_000147155 |
| *Caenorhabditis remanei* | 145 | GCF_000149515 |
| *Caenorhabditis tropicalis* | 79 | GCA_000186765 |
| *Dictyocaulus viviparus* | 161 | GCA_000816705 |
| *Dirofilaria immitis* | 85 | GCA_001077395 |
| *Ditylenchus destructor* | 111 | GCA_001579705 |
| *Elaeophora elaphi* | 1 | GCA_000951195 |
| *Globodera pallida* | 124 | GCA_000724045 |
| *Haemonchus contortus* | 320 | GCA_000469685 |
| *Heterodera glycines* | 82 | GCA_000150805 |
| *Heterorhabditis bacteriophora* | 77 | GCA_000223415 |
| *Loa loa* | 96 | GCF_000183805 |
| *Meloidogyne floridensis* | 97 | GCA_000751915 |
| *Meloidogyne hapla* | 53 | GCA_000172435 |
| *Meloidogyne incognita* | 82 | GCA_000180415 |
| *Necator americanus* | 244 | GCF_000507365 |
| *Oesophagostomum dentatum* | 443 | GCA_000797555 |
| *Onchocerca ochengi* | 95 | GCA_001077375 |
| *Onchocerca volvulus* | 96 | GCA_000499405 |
| *Oscheius* | 117 | GCA_001513535 |
| *Panagrellus redivivus* | 65 | GCA_000341325 |
| *Pristionchus pacificus* | 133 | GCA_000180635 |
| *Romanomermis culicivorax* | 323 | GCA_001039655 |
| *Rotylenchulus reniformis* | 314 | GCA_001026735 |
| *Steinernema carpocapsae* | 86 | GCA_000757645 |
| *Steinernema feltiae* | 83 | GCA_000757705 |
| *Steinernema glaseri* | 93 | GCA_000757755 |
| *Steinernema monticolum* | 89 | GCA_000505645 |
| *Steinernema scapterisci* | 80 | GCA_000757745 |
| *Strongyloides ratti* | 43 | GCA_001040885 |
| *Subanguina moxae* | 90 | GCA_000981365 |
| *Toxocara canis* | 317 | GCA_000951555 |
| *Trichinella* | 49 | GCA_001447505 |
| *Trichinella britovi* | 52 | GCA_001447585 |
| *Trichinella murrelli* | 49 | GCA_001447425 |
| *Trichinella nativa* | 48 | GCA_001447565 |
| *Trichinella nelsoni* | 47 | GCA_001447455 |
| *Trichinella papuae* | 47 | GCA_001447755 |
| *Trichinella patagoniensis* | 50 | GCA_001447655 |
| *Trichinella pseudospiralis* | 49 | GCA_001447445 |
| *Trichinella spiralis* | 64 | GCF_000181795 |
| *Trichinella zimbabwensis* | 51 | GCA_001447665 |
| *Trichuris muris* | 84 | GCA_000612645 |
| *Trichuris suis* | 64 | GCA_000797535 |
| *Trichuris trichiura* | 75 | GCA_000613005 |
| *Wuchereria bancrofti* | 90 | GCA_001555675 |
| Myxozoans | 423 |  |
| *Enteromyxum leei* | 68 | GCA_001455295 |
| *Kudoa iwatai* | 31 | GCA_001407335 |
| *Sphaeromyxa zaharoni* | 174 | GCA_001455285 |
| *Thelohanellus kitauei* | 150 | GCA_000827895 |
| Microsporidians | 166 |  |
| *Anncaliia algerae* | 12 | GCA_000385855 |
| *Edhazardia aedis* | 51 | GCA_000230595 |
| *Encephalitozoon cuniculi* | 2 | GCF_000091225 |
| *Encephalitozoon hellem* | 2 | GCF_000277815 |
| *Encephalitozoon intestinalis* | 2 | GCF_000146465 |
| *Encephalitozoon romaleae* | 2 | GCF_000280035 |
| *Enterocytozoon bieneusi* | 4 | GCF_000209485 |
| *Hamiltosporidium tvaerminnensis* | 13 | GCA_000180835 |
| *Mitosporidium daphniae* | 6 | GCF_000760515 |
| *Nematocida parisii* | 4 | GCF_000250985 |
| *Nematocida sp. 1* | 4 | GCA_000738915 |
| *Nosema apis* | 9 | GCA_000447185 |
| *Nosema bombycis* | 16 | GCA_000383075 |
| *Nosema ceranae* | 8 | GCF_000182985 |
| *Ordospora colligata* | 2 | GCF_000803265 |
| *Pseudoloma neurophilia* | 5 | GCA_001432165 |
| *Spraguea lophii* | 5 | GCA_000430065 |
| *Trachipleistophora hominis* | 8 | GCA_000316135 |
| *Vavraia culicis* | 6 | GCF_000192795 |
| *Vittaforma corneae* | 3 | GCF_000231115 |
| Cryptococcus Yeasts | 185 |  |
| *Cryptococcus albidus* | 21 | GCA_001468955 |
| *Cryptococcus bestiolae* | 24 | GCA_000512585 |
| *Cryptococcus curvatus* | 16 | GCA_001028165 |
| *Cryptococcus dejecticola* | 24 | GCA_000512565 |
| *Cryptococcus flavescens* | 23 | GCA_000442785 |
| *Cryptococcus gattii* | 18 | GCF_000185945 |
| *Cryptococcus laurentii* | 19 | GCA_000738825 |
| *Cryptococcus neoformans* | 19 | GCF_000149245 |
| *Cryptococcus pinus* | 21 | GCA_000512605 |
| Apicomplexans | 1255 |  |
| *Ascogregarina taiwanensis* | 6 | GCA_000172235 |
| *Babesia bigemina* | 14 | GCF_000981445 |
| *Babesia bovis* | 8 | GCF_000165395 |
| *Babesia divergens* | 11 | GCA_001077455 |
| *Cryptosporidium* | 10 | GCA_000831705 |
| *Cryptosporidium baileyi* | 8 | GCA_001593455 |
| *Cryptosporidium hominis* | 9 | GCF_000006425 |
| *Cryptosporidium meleagridis* | 9 | GCA_001593445 |
| *Cryptosporidium muris* | 9 | GCF_000006515 |
| *Cryptosporidium parvum* | 9 | GCF_000165345 |
| *Cyclospora cayetanensis* | 45 | GCA_001305735 |
| *Eimeria acervulina* | 46 | GCF_000499425 |
| *Eimeria brunetti* | 67 | GCA_000499725 |
| *Eimeria maxima* | 46 | GCF_000499605 |
| *Eimeria mitis* | 60 | GCF_000499745 |
| *Eimeria necatrix* | 55 | GCF_000499385 |
| *Eimeria nieschulzi* | 63 | GCA_000826945 |
| *Eimeria praecox* | 60 | GCA_000499445 |
| *Eimeria tenella* | 52 | GCF_000499545 |
| *Gregarina niphandrodes* | 14 | GCF_000223845 |
| *Hammondia hammondi* | 68 | GCF_000258005 |
| *Neospora caninum* | 58 | GCF_000208865 |
| *Plasmodium berghei* | 18 | GCF_000005395 |
| *Plasmodium chabaudi* | 17 | GCF_000003075 |
| *Plasmodium coatneyi* | 28 | GCA_000725905 |
| *Plasmodium cynomolgi* | 26 | GCF_000321355 |
| *Plasmodium falciparum* | 23 | GCF_000002765 |
| *Plasmodium fragile* | 26 | GCF_000956335 |
| *Plasmodium gaboni* | 16 | GCA_000576715 |
| *Plasmodium inui* | 27 | GCF_000524495 |
| *Plasmodium knowlesi* | 23 | GCF_000006355 |
| *Plasmodium reichenowi* | 24 | GCF_000723685 |
| *Plasmodium vinckei* | 18 | GCF_000709005 |
| *Plasmodium vivax* | 27 | GCF_000002415 |
| *Plasmodium yoelii* | 23 | GCF_000003085 |
| *Sarcocystis neurona* | 124 | GCA_000875885 |
| *Theileria annulata* | 8 | GCF_000003225 |
| *Theileria equi* | 12 | GCF_000342415 |
| *Theileria orientalis* | 9 | GCF_000740895 |
| *Theileria parva* | 8 | GCF_000165365 |
| *Toxoplasma gondii* | 69 | GCF_000006565 |
| Oomycetes Yeasts | 2655 |  |
| *Albugo candida* | 33 | GCA_001306775 |
| *Aphanomyces astaci* | 76 | GCF_000520075 |
| *Aphanomyces invadans* | 71 | GCF_000520115 |
| *Hyaloperonospora arabidopsidis* | 78 | GCA_001414525 |
| *Phytophthora agathidicida* | 37 | GCA_001314435 |
| *Phytophthora alni* | 236 | GCA_000439335 |
| *Phytophthora cambivora* | 231 | GCA_000443045 |
| *Phytophthora capsici* | 56 | GCA_000325885 |
| *Phytophthora cinnamomi* | 54 | GCA_001314365 |
| *Phytophthora cryptogea* | 103 | GCA_000468175 |
| *Phytophthora fragariae* | 74 | GCA_000686205 |
| *Phytophthora infestans* | 229 | GCF_000142945 |
| *Phytophthora kernoviae* | 37 | GCA_000333075 |
| *Phytophthora lateralis* | 60 | GCA_000318465 |
| *Phytophthora multivora* | 40 | GCA_001314345 |
| *Phytophthora nicotianae* | 71 | GCA_001483015 |
| *Phytophthora parasitica* | 82 | GCF_000247585 |
| *Phytophthora pinifolia* | 132 | GCA_000500225 |
| *Phytophthora pisi* | 59 | GCA_000751395 |
| *Phytophthora pluvialis* | 54 | GCA_001314425 |
| *Phytophthora ramorum* | 67 | GCA_000336535 |
| *Phytophthora rubi* | 48 | GCA_000687305 |
| *Phytophthora sojae* | 83 | GCF_000149755 |
| *Phytophthora taxon totara* | 56 | GCA_001314925 |
| *Phytopythium vexans* | 34 | GCA_000387545 |
| *Plasmopara halstedii* | 75 | GCA_900000015 |
| *Pseudoperonospora cubensis* | 64 | GCA_000252605 |
| *Pythium aphanidermatum* | 36 | GCA_000387445 |
| *Pythium arrhenomanes* | 45 | GCA_000387505 |
| *Pythium insidiosum* | 53 | GCA_001029375 |
| *Pythium irregulare* | 43 | GCA_000387425 |
| *Pythium iwayamai* | 43 | GCA_000387465 |
| *Pythium oligandrum* | 36 | GCA_001573145 |
| *Pythium ultimum* | 45 | GCA_000387525 |
| *Saprolegnia diclina* | 63 | GCF_000281045 |
| *Saprolegnia parasitica* | 53 | GCF_000151545 |
| Algae | 2097 |  |
| *Ectocarpus siliculosus* | 196 | GCA_000310025 |
| *Saccharina japonica* | 543 | GCA_000978595 |
| *Auxenochlorella protothecoides* | 23 | GCF_000733215 |
| *Chlamydomonas reinhardtii* | 120 | GCF_000002595 |
| *Chlorella pyrenoidosa* | 57 | GCA_001430745 |
| *Chlorella variabilis* | 46 | GCF_000147415 |
| *Chlorella vulgaris* | 37 | GCA_001021125 |
| *Coccomyxa* | 12 | GCA_001244535 |
| *Coccomyxa subellipsoidea C-169* | 49 | GCF_000258705 |
| *Cymbomonas tetramitiformis* | 281 | GCA_001247695 |
| *Gonium pectorale* | 149 | GCA_001584585 |
| *Helicosporidium* | 12 | GCA_000690575 |
| *Micromonas* | 20 | GCF_000090985 |
| *Micromonas pusilla* | 22 | GCF_000151265 |
| *Monoraphidium neglectum* | 70 | GCF_000611645 |
| *Ostreococcus 'lucimarinus'* | 13 | GCF_000092065 |
| *Ostreococcus tauri* | 13 | GCF_000214015 |
| *Picochlorum* | 13 | GCA_000876415 |
| *Trebouxia gelatinosa* | 62 | GCA_000818905 |
| *uncultured Bathycoccus* | 5 | GCA_000259855 |
| *Volvox carteri* | 138 | GCF_000143455 |
| *Chondrus crispus* | 105 | GCF_000350225 |
| *Cyanidioschyzon merolae* | 17 | GCF_000091205 |
| *Galdieria sulphuraria* | 14 | GCF_000341285 |
| *Porphyridium purpureum* | 19 | GCA_000397085 |
| *Heterococcus* | 61 | GCA_000498555 |

**Table S4 | Sequence data information.** Number of reads

Species grouped by databases constructed with Kraken, genome size, assembly used, and taxonomic identification code.

|  | ***Corophium volutator*** | ***Hediste diversicolor*** |
| --- | --- | --- |
| Reads sequenced | 712,510,148 | 638,500,244 |
| 85bp reads (trimmed, merged overlaps, filtered for potential contaminants, parasites, cryptic species) | 262,543,535 | 156,112,296 |
| Number of SNPs (unfiltered) | 28855 | 54654 |
| Number of SNPs (1 per RADtag) | 11649 | 22982 |
| Number of SNPs (1 per RADtag, filtered for missingness and MAF>0.01) | 4870 | 3820 |
| Mean depth per individual per locus | 18.41 | 12.64 |

**Appendix 1 – Erosion of ancestral structure violates assumptions of demographic reconstructions**

Erosion of ancestral genetic structure by human-mediated gene flow adds a layer of complexity to models of demographic history and selection that can bias inferences based on genetic data. Typically, reconstructions of introduction routes from genetic data are inferred from measures of genetic distance, phylogenetic relationships, clustering methods, or approximate Bayesian computation (ABC) (1-2). In *C. volutator* and *H. diversicolor* genetic distance, phylogenetic relationships, and clustering methods all fail to recover a relationship between introduced populations and any particular native population or region. This is typically interpreted as evidence for an admixture origin of introduced populations, which is consistent with historical human-mediated gene flow erasing ancestral genetic structure and precludes inference of introduction routes or demographic history prior to admixture (e.g., 3). These methods do not account for the stochasticity of demographic and genetic processes involved during introduction, and ABC has shown promise in overcoming these challenges by calculating the relative probabilities of competing scenarios defined *a priori* using a large number of simulated data sets and comparing them to observed data (4). However, in systems where populations are genetically distinct and have experienced historical human-mediated gene flow there are several roadblocks to this approach. First, when there are many distinct populations and an absence of direct or phylogenetic information about the relationships between them, the number of possible colonization scenarios increases geometrically (e.g., stepwise colonization of 7 populations from a single source amounts to 7! = 5040 scenarios). While not entirely prohibitive, this decreases the power of ABC to differentiate models. Second, ABC approaches have not been able to distinguish between low levels of gene flow over long periods of time or high levels of gene flow over short periods of time (1), making them inappropriate for formally testing complex scenarios involving temporally variable human-mediated gene flow. Third, and most importantly, if human-mediated gene flow has eroded ancestral genetic structure then extant structure may result from any number of combinations of recent and historical processes, and historical events prior to admixture therefore cannot be inferred from SNPs alone. For these reasons, tests of demographic hypotheses using genetic data may be misleading in systems where human-mediated gene flow is suspected to have influenced connectivity. We therefore refrain from speculating about the specific demographic histories of *C. volutator* and *H. diversicolor*, as it is unclear whether genetic patterns reflect incomplete erasure of ancestral structure or emergent structure that has formed since ballast sediments became defunct.

1. Estoup A, Guillemaud T (2010) Reconstructing routes of invasion using genetic data: why, how and so what? *Molecular Ecology* **19**:4113-4130.

2. Cristescu ME (2015) Genetic reconstructions of invasion history. *Molecular Ecology*  **24**:2212-2225.

3. Darling JA, Bagley MJ, Roman JOE, Tepolt CK, Geller JB (2008) Genetic patterns across multiple introductions of the globally invasive crab genus *Carcinus*. *Molecular Ecology* **17**:4992-5007.

4. Beaumont MA, Zhang W, Balding DJ (2002) Approximate Bayesian computation in population genetics. *Genetics* **162**:2025-2035.
